# 

**Published:** 1896-10

**Authors:** 


### CONTENTS:

SELECTIONS.

Article I.—A Talk on Materia Medica. E. C. Briggs. - - 241
" II.—Teeth of Old Age. - - - - - 248
" III.—The Spitting Habit. - - - - - 250
" IV.—Cancrum Oris. J. P. Shaw, D. D. S., - - 252
" V.—Pains After Extraction of the Teeth. - - 255
" VI.—Empyema of the Antrum of Highmore. Frederick C. Cobb, M. D. - - - - - - - 258
" VII.—Remedial Hypnotism. Dr Ralcy H. Bell. - - 265
" VIII.—Retaining Artificial Dentures in the Mouth. Joseph Spyer, M. D., D. D. S. - - - - 269
" IX.—Pulpless Teeth, Roots, and Their Treatment. Adam Flickinger, D. D. S. - - - - - - 271

COMMUNICATED.

National Association of Dental Faculties. - - - - 274

OBITUARY.

Dr. C. W. Spalding. - - - - - - - 284

BIBLIOGRAPHICAL.

Dr. H. N. Wadsworth. - - - - - - - 285

MONTHLY SUMMARY.

Capping Pulps. - - - - - - - - 286

Masticating. - - - - - - - - - 287

Swaging. - - - - - - - - - 287

A New Method of Doing Bloody Operations. - - - 288

